# Progress in Neurology

**Published:** 1903-11-21

**Authors:** 


### Progress in Neurology.

(*Concluded from page* 118.)

**Dangers of Hypnotics.**—Tirard[4] has recently emphasised the fact that no hypnotic is free from danger. The facts that nausea, loss of appetite, constipation and headache may follow the administration of opium, cardiac depression and occasional excitement the use of chloral, and dulness and depression of spirit the employment of bromide, have long been known, but as each new hypnotic is placed on the market statements are made that no untoward effects follow its use. It is, however, very doubtful whether any hypnotic can be used with impunity. For instance, sulphonal long held its ground as a safe hypnotic, but further experience has shown that it may cause drowsiness, headache and ataxic gait, collapse with rapid and weak pulse, and even death. Again, trional has been found to produce anorexia, vomiting, constipation, and hæmatoporphyrinuria, and in some cases collapse and death. Paraldehyde has such a pungent disagreeable taste that a habit is not easily established, but cases have been recorded showing that its prolonged use produces mental and physical deterioration similar to that which results from chronic alcoholism. Urethane may cause vomiting. Again, hypnotics may be exceptionally dangerous owing to disease. Thus, opium is dangerous in cases of pulmonary engorgement and bronchitis, in delirium tremens and chronic alcoholism; chloral may produce very bad results in cardiac degeneration and dilatation, in hysteria and melancholia.

**Arterial Sclerosis and Nervous Disease.**—Starr,[5] in an interesting paper, discusses the *rôle* played in the etiology of nervous disease by endarteritis; thus in the case of apoplexy four-fifths of the patients will be found to have presented distinct prodromata of the attack. For months or even years before symptoms will be found to have occurred, pointing to disturbance of the functions of the brain. These are very variable in character, the most common are dulness and hebetude, impairment of memory, a sense of perplexity or difficulty in regard to matters which should be easy, irritability of temper and lack of control over emotions. Such symptoms are probably due to malnutrition of the neurones following on arterial disease. Consequently a middle-aged person who complains of such symptoms should not be dismissed with the mere diagnosis of neurasthenia; the condition of his arterial system should be investigated, and if arterial sclerosis be found treatment should be adopted. There are many other diseases in which endarteritis is an important etiological factor. Thus anterior poliomyelitis, bulbar paralysis, and ophthalmoplegia are due either to some infection or to arterial disease, and a similar statement is true of myelitis both of the disseminated and transverse varieties. Erb's syphilitic paraplegia is due to malnutrition of the dorsal region of the spinal cord with consequent descending degeneration, the malnutrition is caused by syphilitic endarteritis obliterating the spinal blood vessels. Senile paraplegia, characterised by a slowly progressive paralysis of the legs with imperfect control of the bladder and rectum, is a condition which occurs after the age of 60, and is due not to primary atrophy of neurones but to arterial obliteration in the lumbar bulb. Combined sclerosis, again, is due to malnutrition of the posterior and lateral columns of the cord consequent on arterial disease. It is probable that a few cases of localised peripheral neuritis, *e.g.*, of the brachial plexus, of sudden onset, without the ordinary cause of cold or strain to explain them, are due to rupture of a vessel in a nerve trunk. These cases are slow in their course, and often chronic, owing probably to the formation of a connective tissue scar within the nerve sheath. Neuralgia, herpes and intercostal neuralgia, especially in old people, may be due to arterial degeneration and consequent hæmorrhage into the ganglia. The forms of treatment which appear to be of the most benefit are such as to produce the establishment of an equable circulation, viz., life in the open air, tepid baths, a diet free from alcohol and rich forms of food, moderate exercise and massage. Drugs such as iodide, chloral, and nitroglycerine, which produce arterial relaxation, are of great benefit, but they should not be of constant use, for it is found that cases of arterial sclerosis by no means have a constant high arterial tension. In exophthalmic goitre the tension is uniformly low probably due to excess of the thyroid secretion in the blood, and it is possible that in many cases of temporary or permanent high tension pulse there may be an arrest of the action of the thyroid gland. And, apart from theoretical considerations, it is found that the administration of thyroid extract in conditions of arterial disease produces decided results. Thus in migraine, and those conditions of high arterial tension associated with vertigo and headache, thyroid extract will in many cases arrest the attack.

**Progressively Developing Hemiplegia.**—In 1875 Erb described a slowly progressive spastic paraplegia dependent on primary bilateral sclerosis of the pyramidal tracts. This observation has been abundantly confirmed in the intervening years. More recently it has been shown, first by Mills, that such primary sclerosis may affect the pyramidal tract of one side only. Another such case, with autopsy, has been published by Mills and Spiller,[6] who conclude that there is a form of progressively developing hemiplegia, usually of ascending type, sometimes triplegia or even quadriplegia, due to primary pyramidal degeneration, which begins on one side, and may extend to the other. According to Patrick[7] some of these cases of "progressively developing hemiplegia" are in reality paralysis agitans without tremor. In that disease the symptoms are, as is well known, at first unilateral, and symptoms other than tremor may be the first to develop. Such a case shows, in the early stage, ache and fatigue on exercise, slowness and clumsiness of movement without ataxy and without paralysis, and a characteristic position of the arm. There may be, in addition, an increase in the deep reflexes. A differential sign of the two affections, apparently, is to be found in the presence or absence of Babinski's toe phenomenon.

**Muscle-tonus and Tendon Phenomena.**—Fraenkel and Collins,[8] by a new instrument, have investigated the relationship between the muscle-tonus and tendon phenomena. The view most generally held is that the tendon phenomena were expressions of the muscle-tonus, hypertonia being, next to exaggeration of the tendon jerks, a symptom of disease of the pyramidal tracts. These ideas were, in general, confirmed by the investigation, though exceptions were found. Thus hypotonia with exaggeration of the tendon reflexes was found in 9 per cent.; and in the cases of hypertonia absent or diminished reflexes were present in 6 per cent. The authors concluded that disease of the posterior tracts of the spinal cord produced hypotonia, whilst disease of the pyramidal tracts caused hypertonia. In discussing this paper Dana said that he had found in cerebral hæmorrhages at first absence of the reflexes and hypotonia on the paralysed side, succeeded after a time by hypertonia and increase of reflexes. If, however, the hæmorrhage was more posterior and involved the sensory sphere, there was more definite and more prolonged absence of reflexes.

**Brown-Séquard's Paralysis.**—Two cases of this affection have been recorded by Warrington,[9] one a unilateral traumatic lesion of the spinal cord, the other a syphilitic softening of the cord affecting one side more than the other. The first case was that of a woman who fell out of a window and fractured the bodies of the fifth and sixth cervical vertebræ. She developed an atrophic paralysis of the right upper limb and spastic paralysis of the right lower limb, with a dissociation anæsthesia of the opposite side of the body, below the third dorsal segment, *i.e.*, an absolute loss of sensation to painful and thermic stimuli, whilst touch and localising power were intact, as was also the muscular sense of position. Recovery was perfect. In the second case, after syphilitic infection, retention of urine and cystitis developed, followed by paraplegia of spastic type, chiefly of the left limb; there was loss of sensation over the right limb, at first of dissociation type, later of all forms of sensation. Under energetic mercurial treatment recovery took place except of the functions of the bladder and rectum. From consideration of these and other cases it appears probable that in man at least most of the fibres conveying deep impressions from muscles pass upwards in the posterior columns on the same side, but that the fibres of the posterior roots which convey other impressions, viz., tactile, pain, and temperature pass directly into the grey matter of the substantia gelatinosa, where they arborise round cells of another neuron, whose axis cylinders cross over to the other side, those subserving temperature and pain passing to the antero-lateral region of the white matter, those conveying tactile sensibility having a wider distribution and perhaps also being represented on the same side.

**Toxic Degeneration of Lower Neurones Simulating Peripheral Neuritis.**—Barnes[10] has recorded seven cases of a peculiar affection which clinically resembles in some respects peripheral neuritis, in others progressive muscular atrophy. It is usually the sequel of acute specific fevers, usually beginning about the second or third week after the febrile condition, and involving the muscles from the periphery to the trunk to a varying extent, but especially causing great atrophy of the hand muscles. After a certain stage, when once definite improvement has begun, relapses are not common, and there is a constant tendency to improvement; the small muscles of the hands are the last to recover. Contractions are rarely developed. The condition is probably one of toxic degeneration of the lower neurones, especially of the motor neurones. Six out of the seven cases recorded recovered, whilst the seventh presented changes supporting the conclusions drawn from the clinical evidence.

**Landry's Paralysis.**—Buzzard[11] has isolated from the blood of a patient who died from Landry's paralysis a micrococcus different from any previously described organism. Previous cases examined by modern methods have in some instances shown no demonstrable lesions, whilst the majority have presented lesions such as can be produced by toxins apart from the microbes themselves. In a few cases the lesions are those of a disseminated or diffuse myelitis or meningo-myelitis, and in some of these organisms have been found. In Buzzard's own case the chief macroscopic change was an increased vascularity of the brain and spinal cord, and the histological changes were slight—some chromatolysis and excentration of nuclei of some of the cells—and toxic in character. The micro-organism found in the blood was a micrococcus, presenting certain resemblances to those of Weichselbaum, Still, and Risien Russell. Cultures injected into rabbits produced a rapidly spreading palsy, and the organism could be isolated in pure culture from its blood.

[4] Lancet, April 11, 1903. [5] Med. Rec., July 4, 1903. [6] Jour. of Nerv. and Mental Dis., July, 1903. [7] Ibid., Aug., 1903. [8] Ibid., July, 1903. [9] Med. Chron., April, 1903. [10] Brain, 1902. [11] Brain, 1903.

